# Supplementary material for: A set of multi-entry identification keys to African frugivorous flies (Diptera, Tephritidae)
Source: Zookeys. 2014 Jul 24;(428):97–108. doi: 10.3897/zookeys.428.7366 (PMC4143993; doi:10.3897/zookeys.428.7366)
Supplement: Supplementary material 6 — Key to Ceratitis [file zookeys-428-097-s006.zip › SF6_ZooKeys_key to Ceratitis/key/SF6_ZooKeys_key to Ceratitis/Media/Html/Ceratitis argenteobrunnea .htm]

Ceratitis (Ceratalaspis) argenteobrunnea Munro


***Ceratitis*** **(*Ceratalaspis*)
*argenteobrunnea* Munro**

*Ceratitis* (*Pardalaspis*) *argenteobrunnea* Munro, 1935: 312.

Body
length: 5.08 (4.85-5.40) mm; wing length: 5.33 (5.25-5.4) mm.

Male

Head.
Antenna yellow to yellow-orange. Third antennal segment three times as long as
second segment. Arista with short hairs over entire length. Frons with short
scattered hairs which are distinctly darker than the frons; convex, in lateral
view not distinctly projecting forwards at antennal implant. Chaetotaxy normal
for subgenus.

Thorax.
Postpronotum yellowish, slightly less orange than centre of mesonotum;
unspotted. Ground colour of mesonotum yellow-orange; mesonotal pattern, median
and dorsocentral spots sometimes loosely connected with each other and with
prescutellar spots (as in holotype). Chaetotaxy normal for subgenus. Scapular
setae black. One anepisternal bristle. Scutellum mainly yellow, sometimes
basally somewhat more whitish; with three black separate markings apically;
basally two dark distinct spots. Subscutellum pale with three dark separate
spots except along dorsal edge where touching.

Legs.
Yellow; setation typical for subgenus, mainly pale especially on femora.
Posterior and posterodorsal rows on fore femur pale.

Wing.
Bands with markings extensively yellow. Banding, marginal band continuous;
cubital band joined with discal band (not free as indicated in original
description); medial band joined with marginal band; crossvein r-m before
middle of discal cell. Crossvein dm-cu posteriorly more outwards than anteriorly.

Abdomen.
Yellow or more brownish. Setation and banding typical for subgenus.

Female

As
male except for the following characters: lower eye margins with darker
colouration. Thorax with inner scapular setae pale. Oviscape almost as long as
abdominal terga 3-6 combined.

(Description
after De Meyer, 1998)
